# Co-Designing an Intervention to Prevent Overweight and Obesity among Young Children and Their Families in a Disadvantaged Municipality: Methodological Barriers and Potentials

**DOI:** 10.3390/ijerph16245110

**Published:** 2019-12-14

**Authors:** Didde Hoeeg, Ulla Christensen, Dan Grabowski

**Affiliations:** 1 Department of Health Promotion, Steno Diabetes Centre Copenhagen, 2820 Gentofte, Denmark; dan.grabowski@regionh.dk; 2 Department of Public Health, Section of Social Medicine, University of Copenhagen, 1599 Copenhagen, Denmark; ulch@sund.ku.dk

**Keywords:** co-designing, design-based research, prevention, childhood obesity, families, municipal health professionals

## Abstract

Design-based research (DBR) is an innovative methodology for co-creation, but potentials, challenges, and differences between methodological ideals and the real-life intervention context are under-researched. This study analyzes the DBR process in which researchers, professionals, and families co-design a family-based intervention to prevent childhood overweight and obesity in a rural municipality. It involves interviews with six key stakeholders in the co-design process. Data were coded and analyzed using systematic text condensation, while the theory of the “social effectiveness of interventions” developed by Rod et al. (2014) was used as an analytical tool for unpacking the co-creation process and exploring methodological barriers and potentials. The DBR approach contributed with a feeling that everyone’s perspective was important, and the professionals got a new perspective on the families’ experiences with healthy living they did not previously consider. We also found that the iterative design process did not fully align with the organizational structures in the municipality or with the needs of stakeholders, leading to friction in the partnership. This study emphasizes the complexity of using an anti-hierarchical approach within a hierarchical context, and the importance of being aware of how the DBR approach shapes the partnership, as well as of how the social dynamics within the partnership shape the design process.

## 1. Introduction

Over the past decades, childhood overweight and obesity became an increasing problem for public health worldwide [1]. The latest reviews found that the effects of interventions to prevent overweight and obesity among preschool children led to small significant effects [2,3,4]. Nevertheless, translating and upscaling randomized controlled trials (RCTs) into real-world settings does not necessarily replicate this effect [5,6]. It is known that involvement of parents in preventive interventions is often challenged by recruitment and retention [7]. Therefore, to improve interventions and better integrate programs into the parents’ sociocultural context, it is vital to engage parents in the development, implementation, and evaluation of obesity prevention interventions [8]. Likewise, involvement of local professionals is essential to obtaining contextual knowledge to inform intervention development and implementation [9]. Collaborative work by professionals in a variety of disciplines, local families, and researchers is key to targeting health disparities in families [10]. To optimize interventions addressing the complexities in prevention of childhood obesity, it was argued that creativity and innovation are key and that processes of intervention development must be iterative and adaptable [11].

Design-based research (DBR) is argued to be a suitable approach to creating innovative programs that address complex challenges [12]. DBR is an increasingly popular way of working toward more inclusive and tailored interventions. The DBR approach is, however, relatively novel in health promotion, particularly in relation to the issue of engaging families and professionals from local communities in co-creating interventions to prevent childhood obesity. One review proclaimed the need for more research on how coalitions influence intervention processes when working with community-based childhood obesity prevention interventions [13]. By understanding such mechanisms, researchers and community partners can strengthen intervention design, implementation, evaluation, and sustainability by responding to local contexts [13]. Contexts are fundamental to intervention development, and the implementation of health interventions and should be seen as a series of generative mechanisms in constant interaction with complex and contingent combinations of events and actors [14,15]. Features of context are intervention-specific, meaning that not every aspect of context is relevant in every case [15]. Poland et al. (2009) distinguished between three key dimensions of social context that impact three levels of health promotion work: the target phenomena, the intervention, and the evaluation of health promotion interventions [14].

The social context of the target phenomena in this case study was presented in another paper based on the initial problem assessment [16]. Therefore, the present paper emphasizes the social context of the intervention development with a specific focus on DBR as the methodology for co-designing an intervention to fit the actual social context of the target phenomena. Analyzing the “context of the intervention” entails analyzing the features of settings that impact intervention design and delivery. This may include how settings are commonly understood, localized determinants of health, making stakeholder interests explicit and understanding power relations, as well as focusing on what the health promoters bring with them to the particular setting [17]. In the present paper, we operationalize the “context of the intervention” to focus on the dynamics and social processes between the diverse institutions in which the intervention is designed and embedded.

We study the social “context of the intervention” by applying the notion “social effectiveness” developed by Rod et al. (2013). We use the focus on social effectiveness as an analytical tool to gain a better understanding of how the social processes of DBR collaboration between researchers and stakeholders evolve throughout the project. Rod et al. (2013) argued that an intervention can only be socially effective if it (1) creates a shared understanding between researchers and professionals, and (2) reconfigures the social relationships between researchers and professionals through processes of “exchange”. Partnerships in intervention development work through the establishment of a reciprocal relationship between researchers and professionals. The process as a whole may be understood as including processes of exchange, which involve the following obligations: to give, to receive, and to reciprocate. Shared understandings should not be understood as identical understandings, and the notion does not imply a complete convergence of perspectives [18].

By drawing attention to the social effectiveness of interventions, it is proposed that intervention researchers pay more attention to the social relations in which they are embedded and with which they interfere.

The objective of the present paper is to analyze how the DBR process of co-design was shaped by the meeting between the fields of health promotion research, municipal professionals, and families and how it affected the social effectiveness of the intervention development. We discuss how certain modes of participation were enabled and certain kinds of facilitation of the process were performed—shaped by the project constellation and through the specific arrangement of the DBR partnership. We also reflect on how existing network and hierarchical structures were affected by the process, as well as on how that impacted the process and the collaboration.

### Theory: Design-Based Research and Innovative Facilitation

Design-based research is a human-centered approach widely used in education research [19,20]. It uses existing research methods and follows established norms for sampling, data collection, and data analysis. Thus, it is not the methods but the goals that set DBR apart from other research genres [21]. The aim of DBR is twofold, as the focus is on designing artefacts and developing theoretical insights [22]. The process is qualified by the continuous input of participants who are co-participants in both the design and the analysis [22,23]. It is argued that, when researchers, professionals, and the target group collaborate in conducting the research and disseminating it, this approach helps to create ownership and commitment on the part of the professionals and target groups [24].

The DBR approach is an iterative process; it typically consists of a needs assessment and an ideation phase, followed by feasibility and pilot tests, before implementing a designed intervention. It is characterized by repeated loops of designing, enactment, analysis, and redesign [22,25]. DBR is context-focused, and a successful innovation is a joint product of the design and the context [19]. To achieve innovative and divergent thinking, it is important to involve a diverse group of participants [25]. The continuous inputs from diverse participants cause the researchers to assume the roles of facilitator, developer, and researcher.

When facilitating innovative processes, the positioning of the facilitators is important. Bakdal (2017) distinguished between four competent facilitator positions: (1) the proactive facilitator is characterized by being instructive; the content is defined, the process described, and relations established in talking and listening positions; (2) the active facilitator is characterized by the participants and the facilitator being equal, despite having different responsibilities. Typically, the facilitator is authoritative and responsible for the process, while the participants are responsible for the content; (3) the reactive facilitator elicits the participants’ own responsibility and prevents the participants from free-wheeling, the idea being that the participants move the process forward, while the dynamics are adjusted or elicited by the facilitator; (4) the passive facilitator acts in a less assertive fashion and is only responsible for being available if the participants express the need for a facilitator. Bakdal stressed the importance of being able to determine which position is most beneficial in what circumstance, as well as to vary the position throughout the process [26].

## 2. Materials and Methods

### 2.1. The Setting of the Case Study

The present manuscript describes the co-creation partnership between the research center Steno Diabetes Centre Copenhagen and a Danish municipality. In the partnership, DBR was chosen as the methodology to generate co-design collaboration between local professionals, families, and researchers. The aim of the project was to design a tailored intervention to support families living in this specific municipality in an attempt to prevent childhood obesity. The municipality is characterized as being rural and disadvantaged. It faces several social challenges, such as having a higher-than-average rate of citizens on social benefits (28%) and children living in poverty (4.6%), as well as a low average life expectancy (78.5 years) and a low average disposable income compared with the average levels across Danish municipalities [27].

The municipality has a high prevalence of overweight and obesity compared with the average rates in Denmark. Among adults, 34% are overweight and 21% are obese [28]. Among pre-schoolers, approximately 11% are overweight and 2.5% are obese [29]. The municipality was, however, interested in creating new innovative solutions to address its social challenges regarding overweight and obesity. Three different municipal units were involved in the DBR project and represented both front-line staff, mid-level managers, and to some degree also the heads of the respective units. The three units focused on “health promotion”, “families and prevention”, and “children and daycare”, and only one of them had healthcare as their main objective.

From the research center, the head of the research team, a senior researcher, and a research assistant were involved. The research center bases its projects on theoretical concepts and methodologies from health promotion, where dialogue, participation, and development of action competence are key components. The DBR approach and the participation of both local families and professionals were seen as prerequisites for designing an intervention tailored to the local setting and generating local ownership [30].

The overall initiative and project organization were conducted in a collaboration between the head of the research team and the head of one of the three units. The project was jointly financed by the municipality and the research center. The established stakeholder groups were intersectoral and included mid-level managers and professionals from all three units, as well as two researchers.

Participatory workshops were used to engage professionals and families. A total of 12 families were involved in family workshops, while 34 professionals were involved in workshops. The project had an 18-month timeline in which to design an intervention and to feasibility and pilot test it among families and professionals.

The authors of the present paper, D.G. and D.H., assumed the triple role of researcher, facilitator, and developer in the DBR project, while author U.C. was not part of the partnership under study. Therefore, U.C. was able to contribute a third-party perspective and played a vital role in the analysis and discussions of findings in the present paper.

### 2.2. Components of the Designed Intervention

The designed intervention included (A) a toolbox with five different tools to help the professionals increase the level of involvement and health competences in families, (B) a “theme night” concept for groups of families with young children arranged in local daycare institutions by professionals from each unit to support and educate families in how to increase family health, as well as to give them opportunities for peer-to-peer reflection, and (C) a “shared care” arrangement consisting of individual family health counseling offered as a collaboration between relevant professionals from each municipal unit involved, including health nurses, dietitians, and day-care professionals. The different professions contribute specific knowledge and perspectives on healthy living, parenting, and family life. Families who participated in a “theme night” but were in need of more support could be recruited for the individual “shared care” intervention.

### 2.3. Method: Interviews with Key Stakeholders

In the present case study, the design process was documented using workshop data, minutes from stakeholder meetings, and other process documents and observation notes. Furthermore, when the project officially ended, interviews on the design process were conducted with six key municipal stakeholders. The interviewees included front-line staff (*n* = 3), mid-level managers (*n* = 2), and heads of units (*n* = 1), and they represented health nurses, daycare professionals, dietitians, and health consultants from the three municipal units “health promotion”, “families and prevention” and “children and daycare”. The key stakeholders were recruited by D.H., who explained the objective of the interviews and promised full anonymization. Four of the interviews were carried out at the municipality, one was carried out at a library, and one was carried out over the phone. The interviews lasted from 30 min to 66 min.

The abovementioned process documents were re-read, and all misunderstandings and friction that emerged during the process were used to inform the interview guide. The interview guide was semi-structured and focused on the project’s purpose, goals, methods, results, and experiences, in relation to the designed intervention and the collaborative process between the professionals and the researchers, as well as among the professionals within the municipality.

All interviews were recorded, transcribed, and coded thematically using systematic text condensation [31]. Our analytical steps were (1) to read the transcripts to get an overall impression and identify preliminary themes, (2) to develop code groups from identified themes and code meaningful units reflecting how the key stakeholders experienced the partnership and co-creation process, (3) to synthesize the units from each code group, to present a recontextualization and to select quotes to illustrate the findings, and (4) to elaborate the findings in a theoretical second-order analysis by using the theory of social effectiveness and facilitation strategies as analytical tools to reveal the experiences of the collaborative DBR process. The transcripts were analyzed using Nvivo 12.0, and all authors discussed the interpretations of the findings. In the discussion, the facilitators of and barriers to the DBR approach to co-creation among different institutions are revealed in three themes based on findings from the analysis.

## 3. Ethics

Because the study involved relatively few key stakeholders, we needed to be very careful to anonymize the interviews. Therefore, we do not link profession, job title, or employment unit to the quotes presented in the result section. The interviewees are presented as Stakeholder 1, Stakeholder 2, and so forth. In addition, names and sensitive information were made unrecognizable in the quotes presented. Before the interviews, how the data would be used and how anonymization would be accomplished were carefully explained, allowing the interviewees to feel comfortable. All interviewees gave their informed written consent prior to the interviews, and the study was approved by the Danish Data Protection Agency (Rec. No.: 2012-58-0004).

## 4. Results

### 4.1. Co-Creation—An Equal and Innovative Partnership

The DBR approach, as a co-design process, was a new experience for the stakeholders, as was the workshops as a method of achieving user involvement. All stakeholders initially described the project as being built on an equal partnership between the three municipal units and the research center. This was in contrast to how projects in the municipality usually began.

*“When we normally run projects in the municipality, it’s always initiated from one department; it’s rare for it to start as an interdisciplinary collaboration. Typically, one or two employees initiate the project and it automatically becomes ‘their project’… So, what was different with this project, was that everyone was called in on an equal basis. […] we managed to put three sectors together and start the project as a partnership.”*

Stakeholder 1

Without talking explicitly about the DBR approach, all stakeholders reported that they were encouraged by the innovative co-creation method brought into the partnership by the researchers. The DBR approach made the partnership feel more equal, as no one had a patent on the definition of health and no intervention was prefabricated.

*“The researchers did not come with a prefabricated idea on how we should do it with these families and these professionals. They have actually been very responsive and emergent. They didn’t know what it would end up like, when we started and in my view that is real co-creation, […] neither researchers nor health professionals think they have a patent on what ‘healthy living’ is—I really like that.”*

Stakeholder 4

In the first phase of the project, the researchers carried out workshops with families as the target group, as well as separate workshops with a broad range of relevant professionals. All workshops were facilitated by the researchers, and the participants were encouraged to present, reflect on, and discuss their different perspectives, experiences, and beliefs in relation to family life and childhood obesity. This contributed to the feeling that everyone’s perspective was important to the design process. All workshop data were coded by the researchers, who presented initial findings to the stakeholder group of professionals prior to the ideation phase. In accordance with the DBR process, the researchers generated preliminary context theories based on the data. For validation, the initial findings and theories were presented to professionals and families in new workshops, where they were encouraged to discuss and reflect on the findings and theories. The stakeholders were very satisfied with this stage of the process, as the findings and theories revealed nuances and dynamics in families’ experiences with healthy living and obesity prevention that the professionals did not think of previously.

*“I think that the entire needs assessment in the first phase with family workshops, where the researchers presented us with the challenges parents really have… Making it apparent what the parents think is the problem, compared to what we think is the problem. And how we could start working with this… I think they [the researchers] did some really good groundwork.”*

Stakeholder 2

*“I do actually think that the researchers have been relatively responsive to the practice in the municipality. […] It’s been a positive thing; I feel that we’ve been listened to.”*

Stakeholder 4

These quotes highlight the fact that the researchers started out by entering a proactive facilitator role, as they were responsible for the process, participant involvement in workshops, and the stakeholder meetings. However, the stakeholders expressed their satisfaction with this initial stage of the project, in that they found the researchers attentive and able to make progress in the process.

The DBR is focused on participant involvement to generate local ownership; however, the researchers’ role as proactive facilitators might also have caused the stakeholders to retrospectively reflect on their perception that the project belonged to researchers.

*“[…] It has always been referred to as the research-project—[…] it’s not the researcher’s project, but that’s what it was called… […] In this way, it became their project and not our project. And that’s not their fault, I think it’s primarily the decision that was made, in the beginning, because there should have been a local project manager down here, while the researchers were collaborators […]. That would have been a better solution.”*

Stakeholder 1

*“It’s simply just… that out of sight, out of mind. Now we had the researchers yesterday, now the researchers are gone. That’s how it went… and we wondered what they [the researchers] were working on in Copenhagen?”*

Stakeholder 2

This illustrates an important paradox; even though all of the stakeholders initially perceived the project as an equal partnership, they nonetheless perceived the project as being owned by the researchers.

### 4.2. Designed Tools and Theory Generation

The stakeholders reported that the project resulted in the design of some useful intervention tools that they did not think of previously. This illustrates successful innovation, viewed as a joint product of the designed intervention and the given context [19].

*“We also got something out of it that we didn’t expect, but which we think is really good. The tools are some of the things […] that we hadn’t anticipated or seen in advance […].”*

Stakeholder 1

*“I think it’s been successful that these things have become useful for regular people.”*

Stakeholder 5

*“I think there’s a lot of good things, the needs assessment, the [researchers] started out making, that was really good, and I also think that the intervention concept, the one we will implement, is good.”*

Stakeholder 2

However, the stakeholders did experience the intervention tools as having been developed and delivered by the researchers. This stresses the researchers’ role as proactive facilitators in the initial stages of the project. The researchers took responsibility for the structure of the DBR process, which meant that the stakeholders needed to assume less responsibility.

This was also evident in the initial workshops in which professionals were encouraged to present, reflect on, and discuss their different perspectives on the issue of childhood obesity and family involvement. The researchers’ facilitation contributed to the creation of some shared understandings among the multidisciplinary professionals involved.

*“I do actually think they [the researchers] came up with a lot of good things—also this whole ‘health understanding’—in relation to the fact that we need a common understanding or framework of ‘healthy living’, what are we really talking about—are we talking about the extremes or are we talking more about everyday life, you know?”*

Stakeholder 2

These findings reveal some strengths of the DBR when establishing a co-creation partnership. However, they also show that when the researchers assumed a proactive facilitator role, the stakeholders felt that the researchers were delivering this stage of the process. This illustrates an exchange practice between the researchers and stakeholders, which seemed to configure a social relationship in a way that created the social effectiveness of the partnership.

### 4.3. Process Confusions and Friction—The Piloting Phase

After the phases of needs assessment and ideation on the DBR process, the project entered a new stage. The stakeholder group decided to try two interdisciplinary intervention elements in addition to the designed tools. The “shared care intervention” was to be designed in detail and pilot-tested on families, as well as tested for feasibility among the professionals. At this stage, the project entered a phase that involved some obstacles, as the collaboration proved more complex than the researchers expected. Several professionals revealed that internal dynamics in the municipality were challenging their interdisciplinary collaboration.

*“It’s not just a coordination between the municipality and the researchers. And that’s what makes it tricky—it’s a coordination between the researchers and three different units in the municipality…”*

Stakeholder 4

*“After all, we also had something in-house, [in the municipality] […] there was something in between that didn’t work 100%.”*

Stakeholder 2

Existing relational issues between the involved municipal units influenced the project and may actually have been enhanced by the co-creation process. Moreover, it became evident that the “shared care intervention” led to confusion about roles and responsibilities between the stakeholders and the researchers. At this stage, the stakeholders expressed having experienced that the researchers started delivering less, and that the process stalled.

*“In fact, I think that [in the beginning] they [researchers] delivered a lot. But then I think that somehow it kind of stalled […] and I think the entire plan was really good, but the problem was—I think the gap between them and us was simply too wide.”*

Stakeholder 2

The workshops and stakeholder meetings became intertwined, because the researchers acted as facilitators, developers, and researchers, which increased confusion about the DBR process among the stakeholders. This is an important reflection, because it made the process more difficult to navigate for the stakeholders. When moving further into the specific design of the “shared care intervention”, the researchers asked for an ideation workshop with the front-line staff from the three units involved. The aim was to focus on how the “shared care” could be designed to be feasible for the diverse group of professionals. To facilitate this specific ideation workshop, the researchers wanted a workshop in which the front-line staff could speak freely about the barriers to and potentials for a “shared care intervention” within the municipality. The researchers wanted only front-line staff to participate and not their managers, as the presence of managers could affect staff members’ ability to speak freely about innovative ideas. This created friction, as the managers at this stage wanted to be part of this workshop.

*“If you invite the employee and make some agreements, and it’s not cleared with the manager and the head—You cannot! That’s what went wrong, because they were very bombastic […]. They [researchers] said that the managers should not participate, but they can’t make agreements with employees without the managers authorizing the resources… So, they made a fatal mistake… You can’t ignore these levels of leadership when you work in a municipality—and that’s what they did…”*

Stakeholder 2

This friction clearly illustrates how the researchers’ triple role as facilitator, developer, and researcher seemed to cause confusion during this stage of the design process. In this case, the researchers were focused on creating an innovative and research ethical workshop and did not realize how that affected the local managers, who saw it as a project meeting rather than a research workshop. This underlines the fact that it was a delicate balance for the researchers to navigate this triple role without creating confusion among the stakeholders.

The participating families felt they benefitted greatly from the research workshops, and the researchers, therefore, suggested a concept and program for the “family theme nights”. In the first test, the researchers functioned as the main facilitators, while some of the professionals facilitated parts of the program related to their professional expertise. The researchers then conducted a small-scale pilot and feasibility study among the families and professionals who attended the theme nights. After adjustments, the researchers tried to transfer more responsibility to the professionals, so they would be able to facilitate the “family theme nights” in the next test. However, friction emerged at this stage, because the stakeholder group asked for evaluations to an extent that the researchers could not deliver before testing the concept further. The researchers could (and would) not conduct evaluations of the concept while they were still the main facilitators, as this would not represent the concept’s feasibility in real life with local professionals as facilitators.

The level of confusion increased as the repeated loops of test and retest in DBR meant that the design of “family theme nights” and the “shared care intervention” could not be finalized until several tests were performed, which challenged municipal planning of the final implementation of the entire intervention. The interviews clearly showed that the professionals felt the researchers were not sufficiently specific about what the finalized intervention would look like and, more importantly, how it should be implemented in the municipality.

*“I couldn’t really get a direct answer—how do we make the implementation plan? … I think that was missing! How is it going to work in a municipal context, right?”*

Stakeholder 4

One stakeholder explained why—when collaborating with a municipality—it is important to be firm in one’s decisions regarding the preferred design and clear about how it should be implemented.

*“I think you have to be steadfast […] when something is initiated in collaboration with a municipality, to be completely firm about what you want and how it should be implemented […]. So, you don’t along the way say, we’ve agreed on this, what do we do then. I think that you need to describe how you think things will be from the outset.”*

Stakeholder 6

This demonstrates that the process in a DBR approach does not always align with the needs and demands of the municipality. The iterative DBR process makes it difficult for researchers to deliver an overall design and detailed implementation plan until elements of the intervention are tested. Indirectly this also shows that shared understandings of the DBR process were lacking at this stage. More specifically, friction that may arise can be interpreted as a matter of failed reciprocity. The stakeholders felt that the researchers were not delivering as expected. Thus, the reciprocal process of exchange failed, leading to a situation in which friction and confusion obstructed the social effectiveness of the partnership.

### 4.4. The Organization and Context of the Project—Reflections on Friction

Several stakeholders expressed that they, in the pilot-testing phase, started to feel that the researchers were not sufficiently familiar with the organization and work procedures in the municipality. One said explicitly that the researchers did not understand how things work in their municipality, as the researchers believed things would automatically be initiated when agreements were made in stakeholder meetings.

*“I’ve experienced that there’s been a very large gap. I’ve experienced that they [researchers] had some kind of belief that after we talked about something, it would happen… The world doesn’t work that way […]—it doesn’t run by itself…”*

Stakeholder 2

The researchers were also frustrated and bewildered regarding this friction, as they experienced that agreements on what to do were not honored by the stakeholders. This illustrates a breakdown in the process of exchange between the researchers and stakeholders.

*“The way they [the researchers] ran the project didn’t show any knowledge of our organization and that has caused some problems along the way.”*

Stakeholder 1

This demonstrates the low level of shared understanding among the stakeholders and the researchers at this stage, which affected the process of exchange. It seemed that structural matters prevented the stakeholders from performing the tests as they wanted to. Several stakeholders explained how it can be difficult to work within a municipality, as projects are established directly into the municipal service in question. This makes it difficult to test intervention elements on a small scale prior to implementation of the final intervention.

This conflict between test and implementation may explain the confusion regarding the stakeholders demanding a detailed implementation plan while the researchers found themselves unable and unwilling to deliver such a plan prior to pilot-testing the intervention elements, in an effort to be loyal to the iterative process of DBR. The researchers were not sufficiently aware on this intertwined relationship between tests and implementation into service and, therefore, not skilled enough to facilitate this stage of the project.

Although decisions were made at stakeholder meetings and supported by the management, the stakeholders still felt incapable of facilitating tests of the new initiatives. The stakeholders lacked stronger facilitation at this stage, while the researchers felt they could not act as strong facilitators, because they did not have the required mandate to get things done in the municipality. Furthermore, the researchers hoped to generate more local ownership by entering a less dominating facilitator role, thus letting the professionals test the elements in a way that made sense in relation to their practice.

The professionals were advised as to how the designed intervention elements could be used, while it was made clear that the professionals could make adaptions or adjustments. The only demand was that the professionals report how they adapted or used the designed intervention elements in practice, so that the researchers could register this information for evaluation purposes. However, this created some friction as well. The stakeholders came up with an internal implementation plan for the “theme nights”, but the researchers felt that the concept was changed so radically that it was no longer based on findings from the needs assessment. Moreover, when the stakeholders attempted to test the “shared care” arrangement, the collaboration between professionals from the three municipal units proved so difficult that it did not really take place as the researchers expected. The researchers’ expectation was that, based on the entire design process, the project would be able to test something more innovative by bringing new perspectives into the field of intervention research in this area. However, the stakeholders did not feel able to deliver what the researchers expected of them.

*“We know that we haven’t delivered what we should—but we also don’t really feel we’ve been able to deliver what was expected…”*

Stakeholder 1

In the interviews, all of the stakeholders revealed frustration that the project and partnership did not develop as expected. Due to the organization of the partnership, this project was not prioritized equally across each unit involved, which naturally challenged the tests of the intersectoral intervention elements, even though some of the stakeholders tried to push for action in units other than their own. Profound barriers were found in the organization and hierarchies in the municipality. Stakeholders explained that they did not have the mandate to facilitate what was decided and, therefore, were unable to deliver what was expected of them.

*“We’ve had to operate something in another area, within another unit. And it’s hard because we don’t have a mandate for it.”*

Stakeholder 1

This illustrates how the iterative process of test–retest–test seemed to be less suitable for the local government structures in the municipality. Nevertheless, the stakeholders felt that, thanks to the project, they became better collaborators internally and developed shared understandings among the involved units.

*“[…] I think we have become better together because in some way, the project has forced us to work together… So, I think we’ve seen each other in a new perspective. That is also development… But I also think that we must realize that it’s actually difficult…”*

Stakeholder 2

## 5. Discussion

### 5.1. The Delicate Balance of Doing Research “with” or “on” Participants

DBR’s central goals of designing artefacts and developing theories are intertwined [19]. In this case, this meant that the researchers attempted to do research both “with” and “on” the different participants in the project, depending on the shifting focus on designing or generating theory. Although all participants were perceived as co-designers, the partnership was established between the researchers and the stakeholders, while the families were merely involved in discussions and had a voice through the workshops and pilot tests. Due to practical circumstances related to the recruitment of families and busy family lives, it proved difficult to involve the families to the extent that they could be part of the partnership and directly involved in the design and research in the DBR process. This meant that the social relationship consisted of the researchers conducting research “on” the target group: the families.

In practice, the DBR process can be unbalanced and end up with an emphasis on either the design process or the theory development [22]. In the present case, the professionals experienced both the theory development and the designed elements as usable in their practice. This is in line with the ideal of DBR, which is that the generated theory must be applicable to practice [32]. This combined focus is a strength in DBR as a methodology. However, when the project ran into barriers, the stakeholders felt the emphasis was too much on conducting research rather than on incorporating the design into practice. If the facilitators are also doing the research, the participants may experience that the focus on conducting research is too strong.

The researchers perceived the stakeholders as equal partners “with” whom they were doing research, and the aim was to involve the stakeholders in the entire DBR process, including both research and design. Paradoxically, our findings on the lack of shared understandings between the stakeholders and the researchers indicated that the stakeholders did not see themselves as being involved in the research part of the DBR project. This indicates that the researchers failed to involve the stakeholders in the research procedures to a sufficient extent. It is important to be aware of the stakeholders’ perceptions on whether the overall goal is research and theory development or more practical design outputs. This is vital if shared understandings are to be obtained and maintained.

### 5.2. The Researchers as Proactive Facilitators of the DBR Process

The initial perception that the partnership was equal changed during the project to the perception that it was solely the researchers’ project. This shift calls for reflection on how the DBR project reconfigured the social relationships between the researchers and professionals.

Participants within a partnership often possess different knowledge and come to be responsible for different areas. DBR acknowledges that professionals are usually too busy and lack the knowledge to conduct rigorous research, while the researchers often are not sufficiently knowledgeable about the complexities of the culture, objectives, and politics of an operating system to effectively create and measure the impact of an intervention [20]. In the DBR literature, it is also argued that the process of going from a problem to a design entails making a move from the empirical level to the heuristic level, which requires that the researchers have fairly good knowledge of existing theories in the area and sound scientific intuition when making a new hypothesis about how the particular problem could possibly be solved [22].

In the facilitation literature, it is argued that when the facilitators have evident knowledge about the process, product, and goal of the project, the proactive facilitator role is often entered, particularly among those new to facilitating innovative processes [26]. In the present case, the researchers’ expert knowledge about DBR, as well as the problem under study, led them to assume the role of proactive facilitators, without thinking about how this affected the co-creation process. In the initial phases, the proactive facilitator role seemed to cause the stakeholders to experience the researchers as delivering the project, which established some social effectiveness. It is argued that, when researchers, professionals, and the target group collaborate, this may create ownership [24]. However, we also know that, as the facilitator takes responsibility for more structure, the participants need to take less responsibility [26]. In the present case, the researchers were responsible for managing the design process, following time schedules, conducting and coordinating the needs assessment, and delivering new knowledge to the participants. In this way, the social relationship was reconfigured, such that the stakeholders ended up being less engaged in the design process and less involved in the research procedures than was intended. When the researchers tried to step back into the position of reactive facilitators, it was experienced as them delivering less; this meant that the processes of exchange were broken, and the social effectiveness stalled. However, although the researchers mostly acted as proactive facilitators, they still failed to facilitate the DBR process and avoid the emergence of misunderstandings and frictions.

If the iterative process becomes so abstract and complicated to navigate that it calls for the researchers to position themselves as proactive facilitators, the researchers must be aware how this might affect the reconfiguration of the social relationships within the partnership. In the present case, the researchers’ eagerness to use DBR as a methodology to co-design an intervention tailored to the local context and to create ownership among the participants apparently resulted in the opposite. When the researchers assumed the role of proactive facilitators to navigate the iterative DBR process with the three municipal units, this move paradoxically ended up increasing the experienced gap between the stakeholders and the researchers, which naturally led to limited social effectiveness and low generation of ownership among the participants.

### 5.3. The (Overlooked) Social Contexts of the Project

Even though the DBR approach is context-focused, we were unable to find specific details in the DBR literature on how different features of contexts should be incorporated into the design process. Understanding the relationship between an intervention and its context is vital to determining how interventions work or fail to work [15]. In the initial stages, it is important to analyze the context and capacity for change, focusing on what kind of support must be present or which barriers exist that must be overcome [17].

Hawe et al. (2009) argued that public health interventions should be viewed as events within complex social systems [33]. That is, an intervention should be seen as a critical event in the existence and history of a complex system, e.g., institutions and communities. In a recent paper, Moore et al. (2019) discussed how a number of priority areas in the field of intervention development and evaluation could be included from this complex systems perspective. Following Hawe et al., they pointed to the importance of focusing on the structure of social ties within bounded social networks [34]. In the present case, the researchers tried to uncover the capacities of the professionals and the context in which they work in the needs assessment. However, this may have been too narrowly focused on the professionals’ daily work practices regarding issues related to childhood obesity and the creation of local solutions, thus overlooking the contextual abilities (and attitudes) of the different stakeholders and professionals to collaborate intersectionally and change existing practices.

It was evident that the stakeholders experienced the hierarchical structures as a barrier to the iterative DBR process. Because DBR is a bottom-up process and by its very nature anti-hierarchical, the approach stands in opposition to the highly fixed hierarchical structures within the municipality. Thus, the researchers had to navigate between methodological ideals and the lived life in the municipality, which challenged the co-design process. This became evident in the phase of pilot tests, when misunderstandings and friction occurred between the researchers and the stakeholders. Despite the involvement of several governing levels (front-line staff, mid-level managers, and heads of units) from the three units, it proved challenging to set up new collaborations across the units. The achievement of testing the designed collaborative intervention elements seemed to be highly dependent on timing within each unit, on unpredictable internal issues between the three units, and the stakeholders’ lack of mandate to “get things done” in another unit. These contextual features are in line with earlier research, showing that, while intersectoral committees without formal authority in other departments may generate ideas, they lack the capacity to ensure implementation [35]. This specific feature of the organizational context of the stakeholders may have been overlooked, as the stakeholders were seen as co-designers in the partnership and not as a genuine target group per se. The researchers tried to conduct research “with” the stakeholders and not “on” the stakeholders, to generate local ownership and tailor the intervention to their needs. However, this meant that the researchers did not see the hierarchies and lack of mandates as barriers to accomplishing the tests of the collaborative intervention elements. In the present case, the researchers clearly lacked awareness of how to combine the anti-hierarchical approach with the hierarchical context in the municipality.

If the researchers perceived the stakeholders as an actual target group, it might have brought more focus on their opportunities for changing practices and provided more support in the tests of intervention elements. However, this could also have established a social relationship in which the stakeholders were researched more “on” instead of “with”, which would be expected to generate less ownership among the professionals. Nevertheless, it is a paradoxical finding that the researchers failed to create genuine stakeholder involvement in the research procedures, while the professionals at the same time were overlooked as an actual target group in the intervention. This underlines that doing research “with” or “on” participants was a delicate balance for the researchers to try to achieve.

The breakdown in the chain of exchange between the stakeholders and the researchers affected the social effectiveness of the DBR partnership between the diverse institutions. Our findings on the importance of the partnership between different institutions are in line with previous results; Korn et al. (2018) highlighted the importance of group facilitation, leadership, and shared understanding to multisector coalition work [13]. The design-based research collective stated that one challenge for DBR researchers involves maintaining a productive collaborative partnership with participants in the research context [19]. Cobb et al. (2013) argued that the extended nature of most design experiments calls for the cultivation of ongoing relationships with professionals. These relationships are sustained by negotiating a shared enterprise, which is typically developed in the long run as researchers consistently demonstrate their personal commitment [32]. Korn et al. (2018) stated that, by understanding coalition mechanisms and by coordinating efforts within and across sectors, leveraging existing resources, and responding to local contexts, researchers and community partners can strengthen their intervention design, implementation, evaluation, and sustainability [13]. Rod et al. (2014) argued that public health intervention research would benefit from thorough consideration of the social dynamics in which public health interventions are embedded and from examining the social effectiveness of intervention programs [18]. Based on our findings, we believe that understanding social effectiveness as a vital contextual feature can add nuance to the features Poland et al. (2009) referred to as the “context of the intervention”. Although the partnership in the present case study was built on DBR as a context-focused approach, the researchers were not sufficiently aware of the different contextual aspects. The researchers overlooked a central aspect of the “context of the intervention”, namely, the specific context of the partnership between the different collaborating institutions. Consequently, the researchers were not sufficiently aware of how the dynamics between researchers, stakeholders, professionals, and families shaped the DBR process, or of how the DBR process shaped the partnership.

## 6. Limitations of the Study

We do not argue that the findings in this case study can be generalized to other projects using DBR as co-design approach. We do, however, believe that the in-depth data and analysis are valuable in exploring some methodological potentials and barriers relevant to other researchers and professionals. It is a limitation to this study that it was not possible to interview all seven key stakeholders involved, as one retired at the end of the DBR process and was not interested in participating in an interview regarding the co-design process. Getting interviews with six out of seven key stakeholders is, however, satisfactory. There is a risk that the stakeholders did not express themselves fully in the interviews, due to the sensitive topics on collaborations and the low number of key stakeholders. This might have increased their concerns for revealing information sensitive to their working environment. This is, however, often the case in case studies of small projects. Another limitation in this study is that the family’s experiences of being involved in the co-design were not included in this study. In future research, we believe that families’ perspective is valuable to strengthen knowledge on how to best involve families in collaborative design processes with professionals and researchers to tailor interventions even better to fit their needs.

## 7. Conclusions

Our aim was to study the DBR as an approach to involve researchers, professionals, and families in co-designing an intervention, tailored to the local context, to prevent childhood overweight and obesity. We found that the DBR approach contributed a feeling of creating an equal partnership between the three municipal units and the research center. The design process resulted in innovative intervention elements and generated new theory, which the professionals found usable for their practice. However, we also found that the iterative DBR process was so unlike the professionals’ normal work processes that the researchers became proactive facilitators. This showed the very delicate balance the researchers had to achieve to navigate the DBR process as facilitators, developers, and researchers. The dual focus on doing research “with” and “on” the participants led to misunderstandings among the stakeholders and researchers. Moreover, the iterative design process did not fully align with the organizational structures in the municipality or with the needs of stakeholders, which led to friction in the partnership—friction that was overlooked as a pivotal contextual feature. This emphasizes the complexity of using DBR as an anti-hierarchical approach within a hierarchical context, like that existing in the municipality under study. Nevertheless, it is important to be aware of how the DBR approach shapes the partnership, as well as of how the social dynamics within the partnership shape the design process. We argue that the notion of social effectiveness is valuable as an analytical tool for unpacking contexts of partnerships. This might support researchers and stakeholders in understanding mechanisms that are vital to maintaining an equal and productive partnership between the participants and institutions involved in a co-design process. Furthermore, we suggest that researchers gain a meta-perspective on how their own role as facilitators impacts the social effectiveness of a partnership and the co-creating process. We believe that the theoretical perspectives and reflections presented in the present paper are important to researchers and practitioners collaborating in co-creation partnerships. Moreover, we suggest that more research be conducted on how to establish and maintain good co-creation partnerships between researchers, stakeholders, and families, in relation to developing sustainable childhood obesity interventions tailored to the local context of families, as well as professionals.
